# Fatigue: For safe patients we need safe nurses

**DOI:** 10.1111/jan.16231

**Published:** 2024-05-10

**Authors:** Alison Steven, Nancy Redfern

**Affiliations:** ^1^ Department of Nursing, Midwifery and Health, Faculty of Life Sciences Northumbria University Newcastle upon Tyne UK; ^2^ Department of Health Sciences University of Genoa Genoa Italy; ^3^ Newcastle upon Tyne NHS Foundation Trust Newcastle upon Tyne UK

In an era where the importance of staff wellbeing has become the focus of many organizational activities and self‐help interventions, and the topic of numerous research studies (Almeida et al., 2024), the impact of staff fatigue in healthcare still seems overlooked. In this commentary we highlight the importance of staff fatigue and its detrimental impact on nurses and patient safety. We argue that staff wellbeing should not focus only on mental health and team support, but that more attention is needed on issues of tiredness, exhaustion or fatigue. Tiredness is heaviness or weakness after physical work, which is relieved by rest. Exhaustion is an inability to respond to emotional or physical stressors and is part of a triad of features alongside depersonalisation and lack of job satisfaction known as burnout (Scholliers et al., 2023). Fatigue is a state of reduced physiological and cognitive performance impacting the ability to do ones job.

## 1 FATIGUE AS A NURSING ISSUE

Public interest in the wellbeing of nurses grew markedly during the covid pandemic. Much focus was on mental health and ways of managing emotional exhaustion and moral injury. Many organizations introduced wellbeing initiatives and psychological support, hoping this would lead to better workforce retention and less staff sickness (Bell et al., 2023). A comprehensive staff psychological support system developed in Wuhan at the start of the pandemic was underused by staff who said they ‘didn't need a psychologist’, they needed ‘more rest without interruption’ (Chen et al., 2020). Thus, while nurses appreciated the public recognition, many asked for something different.

Like Chinese colleagues, UK nurses' wellbeing would improve if we were better rested. But many regard working while tired as simply ‘part of the job’—something health care professionals must accept. Other safety critical industries such as aviation and transport exhibit a different attitude and are required by law to have fatigue risk management systems and strategies to recognize and manage staff fatigue safely. Airline pilots are not allowed to fly when tired. Nurses have the same physiology as pilots and train drivers which begs the question; why are nurses and health care staff not offered the same safety standards?

This may partly relate to education, as nurses may not recognize how tiredness impacts on performance at work. Very few UK hospitals have fatigue on their incident reporting systems or ‘risk registers’, but those that do see a steady stream of drug errors, communication problems, difficulties interpreting data and undertaking practical procedures, all exacerbated by staff fatigue.

Culture may also play a part with nurses often portrayed as angels continuing to work no matter what, and doctors as heroes, keeping going even when fatigued. In some workplaces admitting feeling fatigued is perceived as a weakness, an indication of laziness, and lack of care or respect for patients and colleagues. Therefore, it may be extremely difficult for a nurse to voice feeling tired. Some healthcare organizations forbid napping even during unpaid breaks, and nurses who nap during breaks are chastised and reprimanded, making it difficult to take action to manage fatigue. However understanding of circadian physiology and its impact in performance has grown significantly in the last 20 years, and therefore we believe it is time for a change away from this outdated approach.

## 2 WHY NURSE FATIGUE IS IMPORTANT TO PATIENT SAFETY

Fatigue impacts on nurses themselves and their capacity to deliver care competently and safely. This affects patients, colleagues, teams, health care organizations and ultimately results in financial, societal, and human costs (Cho & Steege, 2021; Querstret et al., 2020; Sutherland et al., 2023). Research demonstrates significant associations between fatigue and reduced nursing performance, including poorer management of patient safety (Cho & Steege, 2021) sometimes with catastrophic safety consequences (Cho & Steege, 2021; Querstret et al., 2020; Sutherland et al., 2023). It is implicated in patient safety incidents and patient mortality (Bell et al., 2023; Cho & Steege, 2021), contributing to increased medication administration and technical errors (Bell et al., 2023; Sutherland et al., 2023). Shift work has a particularly negative effect on fatigue.

Wide ranging research, along with experience from safety critical settings demonstrate the mechanisms that underlie worsening performance as we become more fatigued. These include negative impacts on cognition, mood, logical reasoning, decision making, vigilance, attention, reaction times, and the demonstration of empathy. Fatigue increases risk taking and is shown to produce psychomotor performance equivalent to being above legal alcohol limits (Sutherland et al., 2023).

Furthermore, fatigue is highly detrimental to nurses themselves, increasing the risk of Type II diabetes, cardiovascular disease, a range of cancers, depression, anxiety, burnout and occupational injuries (Cho & Steege, 2021; Querstret et al., 2020; Sutherland et al., 2023). Irregular sleep patterns, poor recovery sleep between shifts and chronic sleep restriction are common problems for nurses (Bell et al., 2023; Cho & Steege, 2021; Querstret et al., 2020). Almost everyone needs 8 hours sleep but a third of healthcare workers report getting insufficient sleep (Sutherland et al., 2023), many are shift workers who suffer the adverse effects of acute sleep loss (Westwell et al., 2021). Furthermore, fatigue has been shown to be consistently associated with sickness absence, lowered performance and mental health issues (Cho & Steege, 2021). Chronic sleep restriction reduces subjective feelings of drowsiness, so we don't recognize that our performance is deteriorating (Sutherland et al., 2023). Driver fatigue is a major contributor to road traffic accidents and deaths, and sadly several nurses and other healthcare workers have died driving home after long shifts or night shifts, with one study reporting 42% of nurses and midwives experiencing a car crash or near miss while driving home tired (Westwell et al., 2021). The potential risks to patient and staff safety are clear.

## 3 WHAT CAN BE DONE

Education and awareness raising is needed both at an individual and systems level—the perception in healthcare that it is solely the individual's responsibility to manage fatigue needs to change. Effective fatigue risk management involves the individual, the team, department, the wider organization and perhaps policy makers. Individuals and teams should understand the need for good ‘sleep hygiene’ and know how to mitigate the impacts of fatigue and shift working. For example, evidence shows that napping can alleviate or mitigate negative consequences of fatigue (Querstret et al., 2020; Sutherland et al., 2023).

Departments and organizations need fatigue risk management systems and processes with predicative, proactive and reactive elements. These might include provision of quiet safe dark places, in or near the workplace, for staff to have a 20–30 min power nap during a shift, and ensuring only vital work is done at night during the 3–6 AM ‘circadian nadir’. For example, some hospitals have changed drug rounds from midnight and 6 AM to 1 AM and 7 AM. Others are introducing into team ‘handovers’ discussion about when people will get their power nap. Rostering systems that are based on circadian physiology can also have a positive impact (Querstret et al., 2020; Sutherland et al., 2023). Furthermore, a few units ask the question ‘could staff fatigue have played a part in this error/near miss’ as part of their incident reporting system and are using the results of this to target nurse education and provision of facilities.

Other safety critical industries are required by law to have fatigue risk management strategies and systems, and these are needed across healthcare (Querstret et al., 2020). Queensland in Australia already has a regulatory requirement for effective fatigue risk management in healthcare (Sutherland et al., 2023). Interestingly this did not require large increases in staffing levels, rather, careful attention to mitigations (such as ensuring facilities for rest and that night shift teams include experienced staff) and the frequency, duration and intensity of work (such as only doing work during the night that cannot wait). This together with good supervision and support from the wider team are reported as having made a positive impact.

### 3.1 Example of locally developed fatigue risk management

**FIGURE 1:**
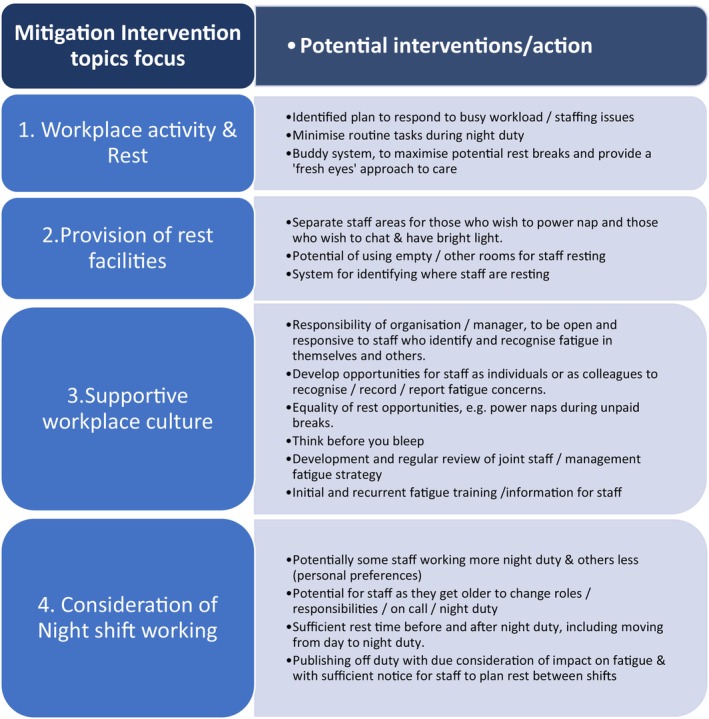
Fatigue risk mitigation topics and suggested actions.

In the UK the authors of this commentary led an action research project working with staff across professions and grades to co‐design a fatigue risk management system in a large maternity unit (Steven et al., 2020). Focus groups were held with a wide range of staff (*n* = 30) to elicit fatigue mitigations suggestions resulting in 37 ideas. Feasible ideas were then ranked by a multiprofessional group (*n* = 14) and those ranked highest then collapsed into 4 ‘mitigation topics’ (see Figure 1).

**FIGURE 2:**
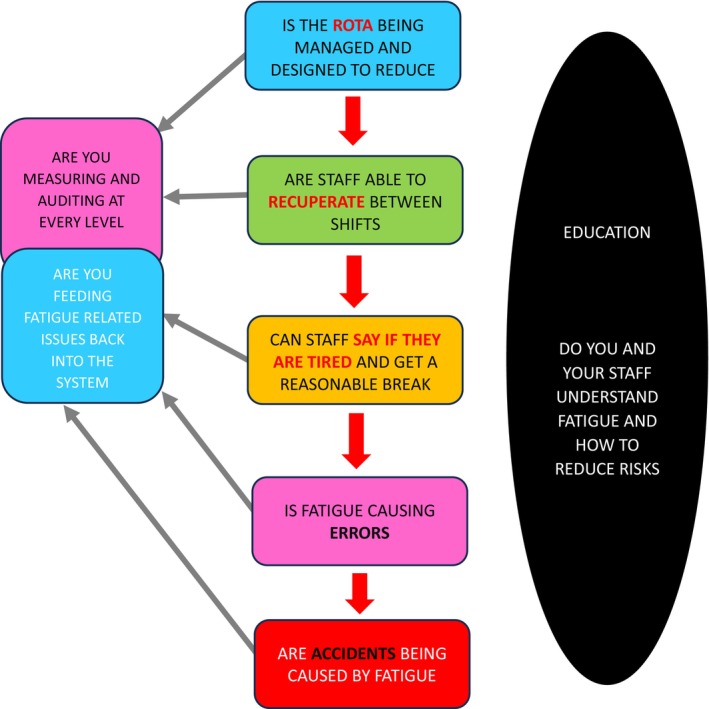
Fatigue risk management in the Maternity Unit—useful questions.

Small groups of staff then focused on each mitigation topic, with the result that staff themselves operationalized actions and strategies thus enhancing engagement, ownership and perhaps the beginnings of a culture change. Actions included: provision of fatigue education and awareness raising; quiet safe spaces with sofa beds and recliner chairs for power naps; changes to rota‐ making arrangements so staff could self‐roster, choosing night shift patterns to better suit their sleep patterns; exploration of a nighttime buddying system; encouragement of only vital work to be undertaken at night; the development of risk management materials (see Figure 2 example) and collaborative writing of a Fatigue Risk Management Strategy. Since the initial action research project ended work has continued with staff being successful in getting fatigue included as a potential factor in the organization's incident reporting system and added to their risk register.

## 4 CONCLUDING COMMENTS

These initiatives show that individual, departmental and organizational changes are possible, but there is also a need for cultural change in the way nurses and the nursing profession perceive fatigue. Fatigue in nurses is widespread but is often overlooked or seen as part of the job (Cho & Steege, 2021). Continuing to work while fatigued by multiple physical, mental and emotional demands should not be seen as self‐sacrifice and martyrdom, but as a risk to our own, and our patients' safety. Nursing staff should not be told they cannot take a nap during breaks or made to feel (explicitly or implicitly) that recognizing and stating that they feel fatigued is a weakness or sign of laziness—sit is in fact a sign of respect for patients, colleagues, and the organization.

A shift in our perception of fatigue is needed along with a change in the way we approach fatigue in nursing. We need to demonstrate that our profession recognizes the importance and impact of fatigue and safeguards those in its care—*To have safe patients we need safe, fatigue‐aware nurses*.

## AUTHOR CONTRIBUTIONS

All authors have agreed on the final version and meet at least one of the following criteria (recommended by the ICMJE*): (1) substantial contributions to conception and design, acquisition of data, or analysis and interpretation of data; (2) drafting the article or revising it critically for important intellectual content.

## FUNDING INFORMATION

The action research project mentioned in this commentary was funded by The Health Foundation (ref number 1211354).

## CONFLICT OF INTEREST STATEMENT

No conflict of interest has been declared by the authors.

## Data Availability

The data that support the findings of this study are available on reasonable request from the corresponding author. The data are not publicly available due to privacy or ethical restrictions.
